# Portable astronomical observation system based on large-aperture concentric-ring metalens

**DOI:** 10.1038/s41377-024-01656-2

**Published:** 2025-01-01

**Authors:** Jianli Wang, Yongting Deng, Chengmiao Wang, Yu Lin, Yeming Han, Junchi Liu, Xiufeng Liu, Hongwen Li, Jan G. Korvink, Yongbo Deng

**Affiliations:** 1 Changchun Institute of Optics, Fine Mechanics and Physics, Chinese Academy of Sciences, 130033 Changchun, China; 2 Institute of Microstructure Technology (IMT), Karlsruhe Institute of Technology (KIT), Hermann-von-Helmholtzplatz 1, Eggenstein-Leopoldshafen, 76344 Germany

**Keywords:** Metamaterials, Astronomical optics

## Abstract

The core advantage of metalenses over traditional bulky lenses lies in their thin volume and lightweight. Nevertheless, as the application scenarios of metalenses extend to the macro-scale optical imaging field, a contradiction arises between the increasing demand for large-aperture metalenses and the synchronous rise in design and processing costs. In response to the application requirements of metalens with diameter reaching the order of 10^4^*λ* or even 10^5^*λ*, this paper proposes a novel design method for fixed-height concentric-ring metalenses, wherein, under the constraints of the processing technology, a subwavelength 2D building unit library is constructed based on different topological structures, and the overall cross-section of the metalens is assembled. Compared to global structural optimization, this approach reduces computational resources and time consumption by several orders of magnitude while maintaining nearly identical focusing efficiency. As a result, a concentric-ring metalens with a designed wavelength of 632.8 nm and a diameter of 46.8 mm was developed, and a quasi-telecentric telescope system composed of aperture stop and metalens was constructed, achieving high-resolution detection within a 20° field of view. In the subsequent experiments, the unique weak polarization dependence and narrowband adaptability of the meta-camera are quantitatively analyzed and tested, and excellent imaging results were finally obtained. Our work not only ensures the narrowband optical performance but also promotes the simplicity and light weight of the metalens based telescopic system, which further advances the deep application of large-diameter metalenses in the field of astronomical observation.

## Introduction

Metalenses modulate the light field with subwavelength structures, offering numerous advantages such as lightweight design, ease of integration, and high controllability^1,2^. Currently, the applications of metalenses are predominantly concentrated in miniature optical imaging systems, including micro-robots, endoscopes, and portable microscopes^3–6^. However, their application in fields requiring large aperture sizes, on the order of centimeters or even decimeters, such as visible light telescopes^7,8^, thermal infrared detection^9,10^, and optical remote sensing^11^, is still somewhat constrained by the associated design and manufacturing costs.

In the usual sense, metalens is assembled from an array of subwavelength nano-posts with fixed heights. Various waveguide modes are generated by the interaction between the local light field and nano-posts with different cross-sectional shapes, and the multi-dimensional independent modulation of amplitude, phase, polarization and other parameters is realized^12,13^. Owing to their outstanding multifunctional modulation capabilities, metalenses based on nano-posts arrays play an irreplaceable role in applications such as ultra-high-resolution microscopy^14–16^, broadband achromatic imaging^17–21^, holography^22–24^ and polarization multiplexing^25–27^. However, with the increase of the diameter of the building-block-array metalens to the order of 10^4^*λ*–10^5^*λ*, the number of nano-posts contained in the metalens reaches 10^8^ to 10^11^, the fly in the ointment of long processing cycle and high processing cost caused by the vast amount of layout data gradually appeared. Correspondingly, concentric-ring metalenses with fixed height inherit the advantages of building-block-array metalenses in polarization-independent focusing scenarios^28–30^. With significantly fewer nanostructures, the processing costs of the concentric-ring metalens are substantially reduced, while its mechanical stability is significantly improved due to the nearly infinite extension of nano-rings along the tangential direction of the metalens, making it a more competitive and innovative choice in the application of visible light telescoping.

The prevalent structural design methods for concentric-ring metalenses are based on global structural optimization. Core optimization methods such as topology optimization^31–34^, genetic algorithm^21,35,36^ and deep learning^37–41^ are employed to construct targeted optimization models, aiming to obtain optimal solutions that meet the requirements of the manufacturing process. Such a technical approach can achieve concentric-ring structures with excellent optical performance, but when the design requirement with diameter reaching thousands of wavelengths or more, it imposes extremely high demands on computational power, and the computational process also incurs tremendous time costs. To solve this problem, this paper utilizes 2D subwavelength building units to splice the cross-section of the concentric-ring metalens, wherein the unit structures are classified based on different numbers of nano-rings, and their ring width and ring spacing are fine-adjusted. The method compresses the computational power consumption and time cost by several orders of magnitude while ensuring that the focusing efficiency is almost no less than the global optimization result^30^. The resulting 2D building units are arranged according to the target phase obtained by geometric optical optimization to form the overall cross-section of the concentric-ring metalens.

Typical visible metalens telescope systems with a centimeter-scale aperture can theoretically only achieve monochromatic near-diffraction-limited focusing under normal incidence^7,8^. Meanwhile, optical systems that realize full-field high-resolution imaging using a stop-metalens combination scheme typically feature apertures on the order of 10^3^*λ*^42,43^. In this study, the aperture of our metalens has been extended to nearly 10^5^*λ* based on the concentric-ring structure design, and the high-resolution imaging performance across the entire field of view (FOV) can be preserved through the precise design of the overall stop-metalens combination system. To the best of our knowledge, this is the largest aperture full-field high-resolution metalens telescope system to date. Comparisons of performance parameters between this work and other notable related literature are presented in Supplementary Information S1.

Ultimately, a 46.8 mm diameter metalens designed using this method is integrated with an aperture stop to construct a quasi-telecentric meta-camera with high resolution (MTF ≥ 0.4@46 lp/mm) within FOV of 20°. Based on the aforementioned efforts, the aperture of concentric-ring metalenses working in visible can be extended to the centimeter scale under the premise of low cost, ease of manufacturing, and high performance, which further enhances the practical competitiveness of metalenses with apertures on the order of 10^4^*λ*–10^5^*λ* in applications such as visible light panoramic imaging and portable astronomical observations.

## Results

### Design method

Metalens imaging for infinite-distance targets commonly utilizes the hyperbolic phase for the target phase, aiming to achieve diffraction-limited focusing of normally incident parallel light, as shown in Eq. (1),

1 $${\varphi }_{0}(r)=\frac{2\pi }{\lambda }(\sqrt{{r}^{2}+{F}^{2}}-F)$$

where *λ* is the wavelength, *r* is the radial coordinate, and *F* is the focal length.

**Fig. 1:**
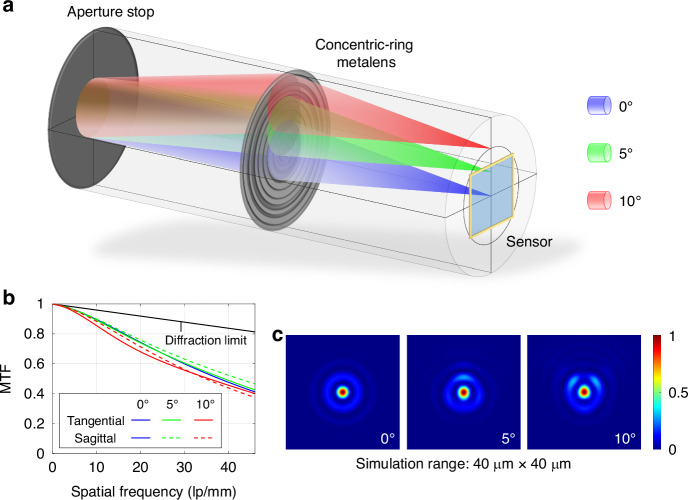
Structure diagram, MTF and normalized PSF of the integrated telescopic system. **a** Structure diagram of integrated telescopic system with aperture stop and concentric-ring metalens; **b** MTF of the telescope based on geometric optics optimization; **c** normalized PSF for incident angles of 0°, 5° and 10°

Such designs are highly sensitive to off-axis aberrations, making it difficult to achieve wide-field high-resolution imaging. Therefore, the Chevalier Landscape lens^6,42,43^ is adopted as the design basis for the optical system, that is, introducing a front aperture stop to limit the edge ray aberration. The aperture stop is positioned approximately at the front focal plane of the metalens to achieve image-side telecentricity, as shown in Fig. 1a, which is conducive to limit the chief ray angle, thereby reducing the sensitivity of Point Spread Function (PSF), Modulation Transfer Function (MTF) and Relative Illuminance (RI) to the field angle *θ*^44^. Building upon this, the target phase is expressed as an even-order polynomial, as shown in Eq. (2),

2 $${\varphi }_{0}(r)=\frac{2\pi }{\lambda }{\mathop{\sum }\limits_{s=1}^{6}{a}_{s} \left(\frac{r}{R}\right)}^{2s}$$

where *R* is the lens radius and *a*_*s*_ represents the coefficients of the *s*-order terms to be optimized.

Under the condition where the imaging-side resolution matches the cut-off frequency of the detector (MTF ≥ 0.4@0.5*D*_*pixel*_^−^^1^) across the entire FOV, the key parameters such as the coefficients of even-order terms of the target phase, the distance between the stop and the metalens, and the back focal length are optimized based on geometric optics theory with the objective of maximizing the entrance pupil diameter. The MTF of the optimized optical system is shown in Fig. 1b, and PSF is shown in Fig. 1c. See Supplementary Information S2 for detailed optimization results.

After obtaining the geometric optics optimization results of the target phase, it is necessary to construct the concentric-ring structure to achieve wavefront modulation approaching the target phase. Benefiting from the central symmetry of the optical system, the design of the concentric-ring structure can focus on the 2D cross-section along the radius of the metalens. The 2D nano-ring cross-section inherits the weak coupling characteristics of the 3D nano-posts in building-block-array metalenses, so the design work for concentric-ring structures can be further simplified into the design of 2D unit structures. At the same time, the approximation of truncated waveguides remains applicable to 2D unit structures, which determines the insensitivity of its modulation characteristics to the incident angle, ensuring the logical self-consistency of the arrangement of 2D building units guided by the optimization results in which multiple fields of view are considered simultaneously. In this work, the 2D units were categorized into different groups based on the number of internal nano-rings, and the structural design with quasi-continuous scanning of size parameters was conducted under the conditions permitted by the manufacturing process, further enhancing the focusing efficiency compared to existing static discretized design variable traversal methods^30^.

**Fig. 2:**
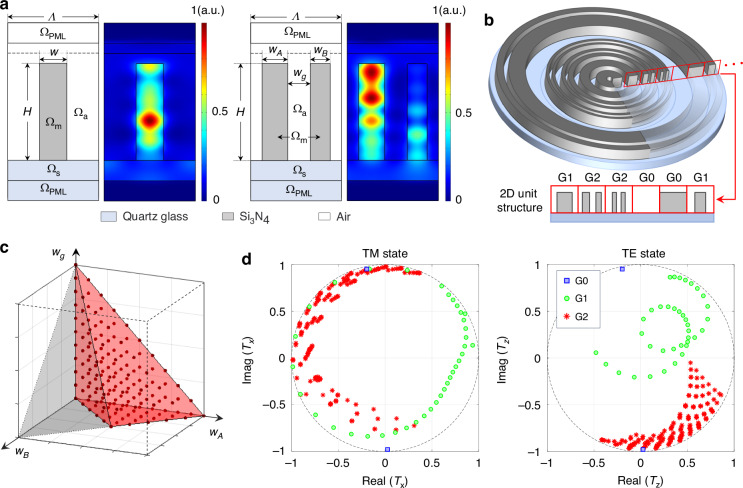
Simulation diagram, sketch demonstration, parameter space and complex transmittance of the metalens. **a** Simulation diagram of single-ring structure (G1) and double-ring structure (G2) and their energy density distributions; **b** whole structure of the concentric-ring metalens obtained based on 2D unit splicing; **c** parameter space diagram of G2 structure, where the red region and the scattered points inside represent the available value space and actual sampling points, respectively, the range of the entire cubic region is [*w*_*min*_, *Λ*-3*w*_*min*_]^3^; **d** complex transmittance of different structure groups for TM wave and TE wave

In the 2D unit design model, equal-intensity TE and TM waves with a wavelength of 632.8 nm are incident from the substrate side. The complex transmittance of both polarizations is detected above the nano-ring cross-section. Silicon nitride is employed as the dielectric material for the construction of the nano-rings, whose refractive index is set to 2.005. The feature size (*w*_*min*_) of the nano-rings and the air gaps are set to 120 nm, so the number of nano-rings within a unit cannot exceed 2 when the unit width varies in the subwavelength range. Figure 2a is the schematic diagram of the simulation models of the single-ring group (G1) and the double-ring group (G2), where *Λ* is the undetermined unit width; *H* is the structural height; Ω_a_, Ω_m_, Ω_s_ and Ω_PML_ are the air domain, the material domain, the substrate domain and the perfect matching layer, respectively; and the edges of both sides of the simulation domain are set as continuous periodic boundary conditions. In addition to the two main groups mentioned above, the all-air structure and the all-material structure constitute the ringless group (G0), serving as supplementary structures to maximize the range of phase modulation. To ensure weak coupling approximation and manufacturability, the width of the air gap at the edge of the units in G1 and G2 must be greater than *w*_*min*_ /2, so the modulation characteristics of G1 can be obtained by traversing the 1D parameter space of *w*∈[*w*_*min*_, *Λ*-*w*_*min*_]. Due to the equivalence of the two nano-rings, the structural parameters of G2 can be constrained to *w*_*A*_ < *w*_*B*_, so the 3D parameter space for G2 can be described as *w*_*A*_∈[*w*_*min*_, *Λ*-3*w*_*min*_], *w*_*B*_∈[*w*_*min*_, *w*_*A*_], *w*_*g*_∈[*w*_*min*_, *Λ*-*w*_*min*_ -*w*_*A*_-*w*_*B*_], as illustrated in Fig. 2c.

To determine the optimal values for the underlying parameters *Λ* and *H*, simulations are conducted within the specific parameter space domain, with the primary objective of maximizing the focusing efficiency of the optical system. The focusing efficiency is defined as the ratio of the energy within five times the Full Width at Half Maximum (FWHM) of the diffraction-limit focal spot to the total transmitted energy. Taking into account both the processing difficulty and the focusing performance, the unit width is ultimately set as *Λ* = 600 nm, and the structural height is *H* = 960 nm. See Supplementary Information S3 for details. Under this underlying parameter setting, the width sampling interval of the nano-rings is set to 5 nm, and the modulated complex amplitudes of TE and TM waves for the three structural groups are shown in Fig. 2d.

It can be observed that the 2D building unit exhibits distinctly different complex transmittances for two polarization states. Therefore, it is almost impossible to achieve strict target phase matching for both polarization states simultaneously by using one structural arrangement. More specifically, strip-shaped nanostructures induce more complex multiple resonance modes for the light polarized along them, resulting in more pronounced backscattering energy loss and an inability to cover the 2π phase modulation range for TE waves. Under these conditions, it is necessary to introduce different phase correction constants to TE and TM waves on the basis of the common target phase in Eq. (2), so that the optimal phase matching for two polarization states can be achieved simultaneously by adjusting their phase constant difference Δ*φ*. Therefore, Δ*φ* should be optimized as a primary parameter in the unit arrangement process. For any assumed Δ*φ*, the 2D building units are arranged based on the principle of maximizing the complex transmittance projections for both polarization states. For the *m*-th unit, the simulated complex transmittances (${\widetilde{T}}_{\text{TM}}$,${\widetilde{T}}_{\text{TE}}$) of the two polarization states are projected onto the target complex transmittance, and the equal-weighted sum of the two projection values *γ*_*m*_ should be maximized, as shown in Eq. (3),

3 $$\begin{array}{c}{\rm{Max}}\,{\gamma }_{m}={\rm{real}}\{{\tilde{T}}_{{\rm{TM}}}\exp [-i{\varphi }_{0}(m\Lambda -\varLambda /2)]\}\\+\,{\rm{real}}\{{\tilde{T}}_{{\rm{TE}}}\exp [-i{\varphi }_{0}(m\varLambda -\varLambda /2)-i\varDelta \varphi ]\}\end{array}$$

**Fig. 3:**
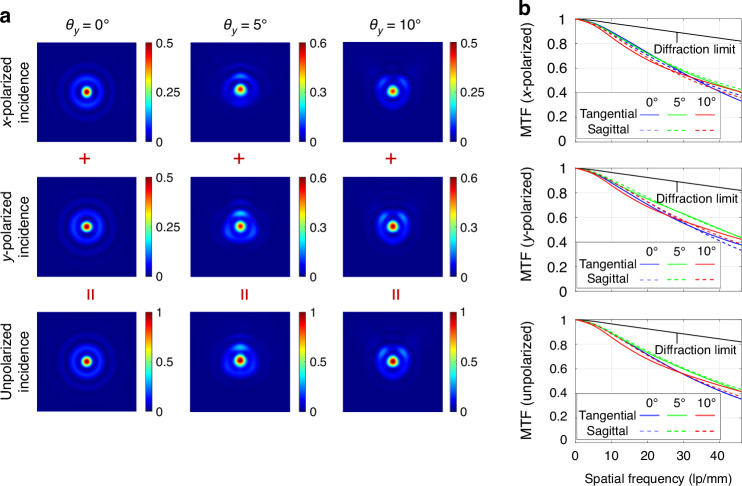
Simulated focusing results of the optimal concentric-ring structure. **a** PSF for different incident polarization states and incident angles, where all subgraphs are normalized according to the subgraphs of non-polarized incidence at the corresponding incident angles and the simulation ranges are 40 × 40 μm; **b** MTF curves for different incident polarization states

Based on this principle, the overall structure and the focusing efficiency of the metalens only depend on the selection of the phase difference Δ*φ*. Unlike building-block-array metalenses that can achieve strict polarization-independent modulation, concentric-ring metalenses, while capable of achieving overall polarization-insensitive imaging, are inevitably subject to polarization dependence due to the strip-shaped nanostructures in the local perspective. Thus, the polarization conversion introduced by the strip-shaped nanostructures should be carefully considered in the phase difference optimization with the goal of maximizing the focusing efficiency. More specifically, the calculation process of the focal plane intensity distribution needs to be performed separately for different incident angles (*θ*_*y*_) and polarization states of the incident light. After traversing the phase difference Δ*φ* and obtaining its optimal value, the simulated focusing performance of the optimized concentric-ring structure is illustrated in Fig. 3. The detailed calculation method is provided in Supplementary Information S4.

Due to the different complex transmittances of the metalens for radial and tangential polarizations, subtle differences in the output light intensity distribution will exist depending on the incident polarization state. The incidence of non-polarized light is equivalent to the equal-intensity incidence of x- and y- linearly polarized lights with no fixed phase relationship between them, and the final intensity distribution is the incoherent superposition of the two intensities. By comparing the last row of Fig. 3a with Fig. 1c, and Fig. 3b with Fig. 1b, respectively, it can be observed that the simulated focusing performance of the concentric-ring structure is almost identical to the results of geometric optics optimization. The imperfection in light field modulation due to the non-strict matching of dual-polarization complex transmittances is primarily manifested in a slight loss of focusing efficiency, which will hardly cause the dispersion of PSF and the reduction of the MTF curve. After the optimal value of Δ*φ* that maximizes focusing efficiency is determined, the whole 3D structure of the concentric-ring metalens can be finally obtained by rotating the arranged nano-ring cross-section around the optical axis for one complete revolution.

### Fabrication and optical performance test

**Fig. 4:**
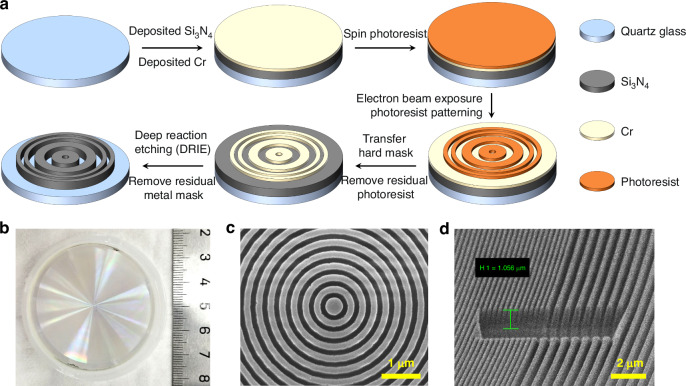
Fabrication process, physical image and scanning electron microscopy views of the metalens. **a** Processing flow diagram of the concentric-ring metalens; **b** physical image; **c** scanning electron microscopy top view of the central region; **d** scanning electron microscope oblique view of eccentric region

The diagram of the process flow and the final characterization results of the concentric ring structure are shown in Fig. 4. See the “Materials and methods” section for a detailed description of processing.

**Fig. 5:**
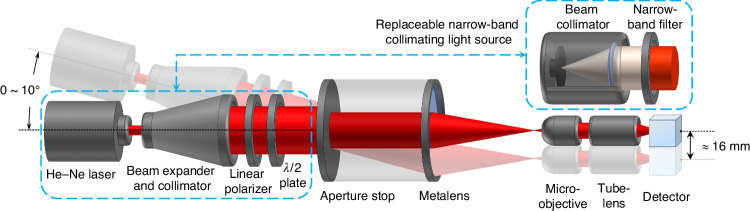
Diagram of the optical system used for PSF testing

After the metalens samples were obtained, we conducted PSF tests on the meta-tube packaged with the concentric-ring metalens and an aperture stop with a diameter of 18.2 mm under different FOV, polarization states and bandwidth. The diagram of the test system is shown in Fig. 5. The He-Ne laser with a wavelength of 632.8 nm enters the meta-tube after beam expansion and collimation, and is focused on the back focal plane. To verify the weak polarization dependence, a detachable linear polarizer and a *λ*/2 plate are inserted behind the monochromatic collimating light source to generate x- and y-polarized light. The micro-objective, tube-lens and detector (MER2-2000-19U3C-L, Daheng Co. LTD) together constitute a 40× microscope system for magnifying the focal spot of the meta-tube. Since the object-side FOV of the microscopic system is much smaller than the image-side FOV of the meta-tube, the position of the microscopic system needs to be adjusted accordingly when the light source is deflected to test PSFs at different incident angles. In addition, a white light collimator and a 5 nm bandwidth filter form a replaceable non-polarized narrowband collimated light source, which is used to test the narrowband performance and estimate the resolution in actual observation scenarios.

**Fig. 6:**
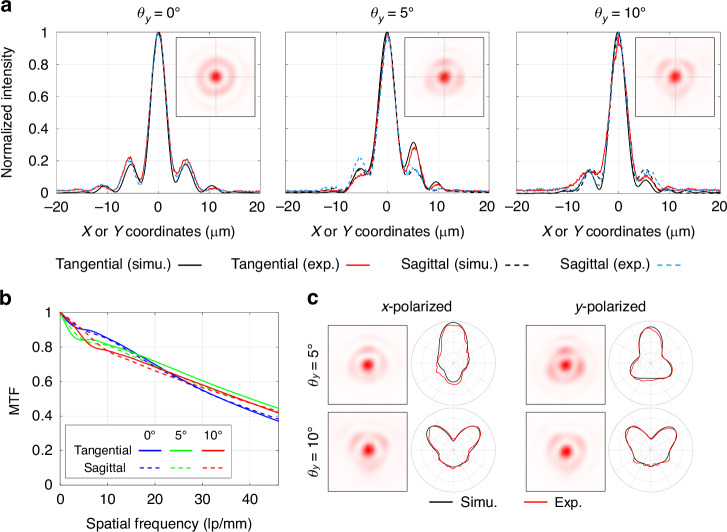
Test results of the monochromatic focusing performance of the meta-tube. **a** Non-polarized PSFs and their normalized intensity curves in tangential and sagittal cross sections; **b** MTFs curves calculated from the experimental PSFs; **c** PSFs and the corresponding relative intensity distributions of the first-order side lobes under different polarization states and oblique incidence angles where all the intensity curves in polar diagrams are linearly scaled to a mean square value of 1 and the maximum radial coordinates are *r* = 2

Figure 6 shows the test results of the monochromatic focusing performance of the meta-tube. In Fig. 6a, the normalized PSFs within the tangential and sagittal sections under the condition of non-polarized light incident are shown, and the subgraphs in the upper right corner of each figure depict the original appearance of the measured focal spots. The FWHMs of the focal spots under different FOVs are shown in Table 1. It can be seen that the main peak widths of the experimental and simulated results are nearly identical, and the trends in the variations of the measured and simulated intensities of the side lobes are noticeably similar. Building upon this, the corresponding MTFs were calculated based on the measured PSFs, as shown in Fig. 6b. The MTFs at cut-off frequency for different view angles are approximately 0.4, maintaining a high level of consistency with the geometric optical design expectations depicted in Fig. 1b. The minor oscillation at the low-frequency region of the MTF curve is associated with the dispersion of light from non-required diffraction orders (primarily the direct transmission of the 0-order diffraction) onto the image plane, which has little impact on the overall trend of the MTF curve after being manually removed.

**Table 1:** FWHMs of the simulated and experimental focal spots under different FOVs

	0°		5°		10°	
	Tangential	Sagittal	Tangential	Sagittal	Tangential	Sagittal
Simu.	3.26	3.26	3.77	3.63	3.51	3.48
Exp.	3.55	3.45	3.68	3.45	3.80	3.69

Furthermore, a targeted test on the weak polarization dependence of the meta-tube was conducted to verify the accuracy and rationality of light field calculation with different polarization in the design stage. The simulation results show that as the oblique incidence angle increases, both the relative intensity distribution of the first-order side lobe and the total spot intensity gradually exhibit correlations with the polarization states of the incident light. In terms of side lobe analysis, the intensity distribution aligns extremely well with simulation results and exhibits specificity for different polarization states. As shown in Fig. 6c, the incident polarization state can be even reversely determined according to the intensity distribution of the side lobe. In the polar plot, the angular coordinate represents the azimuth angle of any point on the first-order side lobe relative to the spot center, while the radial coordinate represents the relative intensity of the first-order side lobe at that azimuth angle. Specific details on side lobe appearance measurement and calibration are provided in Supplementary Information S5. In terms of the total intensity of the focal spot, simulation results indicate a spot intensity ratio of 0.98 between x- and y-polarized incidence at *θ*_*y*_ = 5°, and no identifiable difference between the two is observed in the corresponding measurement. At *θ*_*y*_ = 10°, the difference in spot intensity for x- and y-polarized incidence becomes more pronounced, with simulations suggesting an intensity ratio of 0.75. The corresponding experimental results are approximately 0.69. Both the relative intensity distribution of the first-order side lobe and the total intensity ratio are highly consistent with the simulation results, which fully proves that the prediction of the weak polarization dependence of the concentric-ring metalens is accurate and reasonable.

**Fig. 7:**
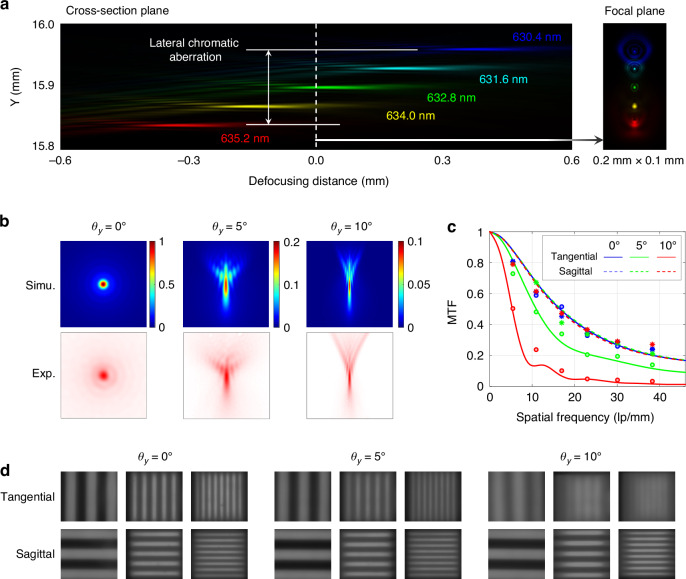
Simulation and experimental results for PSF and MTF of the metalens. **a** Simulated diagram of the lateral chromatic aberration in the cross-sectional plane and focal plane, with an oblique incidence angle *θ*_*y*_ = 10°; **b** comparison of simulated and experimental results of narrowband PSF, the corresponding sampling ranges are 40 µm, 80 µm, and 160 µm at incident angles of 0°, 5° and 10°, respectively; **c** comparison of tested contrast ratios of targets with different spatial frequencies and simulated results of narrowband MTF; **d** images of three targets with different field angles, whose spatial frequencies are 10.9 lp/mm, 23.0 lp/mm and 38.3 lp/mm, respectively

After thoroughly verifying the monochrome design results, the incident light source was replaced from He-Ne laser to red light with a central wavelength still at 632.8 nm and a bandwidth of 5 nm so as to more accurately estimate the focusing performance in actual observation scenarios. For the meta-tube with a centimeter-level entrance pupil diameter discussed in this article, lateral chromatic aberration is a more significant factor affecting image quality compared to axial chromatic aberration. The simulated diagram of the lateral chromatic aberration is shown in Fig. 7a. The comparison between experimental and simulated results of narrowband PSFs is illustrated in Fig. 7b. Further, we replace the pinhole used for the equivalent point target in the narrowband collimating light source with different resolution targets to test the resolution capability for objects at infinity. The contrast ratio testing results with different spatial frequencies are indicated in Fig. 7c, showing great consistencies with the MTF curves calculated from simulated PSFs. The three target images with spatial frequencies of 10.9 lp/mm, 23.0 lp/mm and 38.3 lp/mm are shown in Fig. 7d. As can be seen, the sagittal contrast ratio will not be further affected by lateral chromatic aberration on top of axial chromatic aberration, which largely ensures that the observed resolution of natural scenes will not have a serious deviation from the design results. In addition to the tangential direction of the edge field of view, the high spatial frequency targets in most positions are intuitively distinguishable, demonstrating the rationality of using a monochromatic designed metalens for nano-bandwidth imaging in this work.

**Fig. 8:**
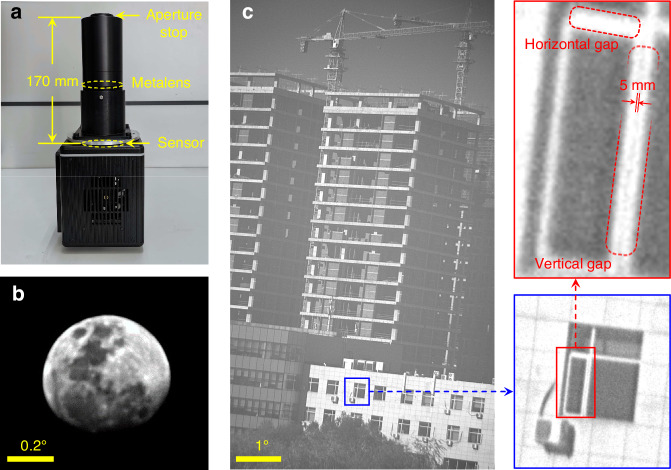
Meta-camera and the imaging results. **a** Physical photo of the meta-camera; **b** enlargement of the moon image in the central field of view; **c** image of the distant building and its local magnification

Finally, the meta-tube, a narrowband filter with a bandwidth of 5 nm and the detector were reassembled into a meta-camera for the real scene imaging test. The physical photo of the prototype is shown in Fig. 8a. We imaged the moon using the central region of FOV. The resulting image is shown in Fig. 8b. During this process, an image edge enhancement method was utilized to slightly increase the contrast of the image, making the surface details of the moon more abundant and apparent. Figure 8c shows an image result of a distant building target, which is about 300 m away from the meta-camera. It is worth noting that in the zoomed-in portion of the image, the meta-camera can effectively resolve the vertical window frame gap with a width of 5 mm, and the corresponding resolution angle is approximately 13 µrad, demonstrating the significant resolution capability for single-line targets. Meanwhile, the resolution capability of the meta-camera for horizontal gaps is weaker than that for vertical ones, which is attributed to a small amount of lateral chromatic aberration present in the local image formed under a vertical field angle of approximately 4°. This phenomenon qualitatively reaffirms the earlier analysis regarding lateral chromatic aberration.

## Discussion

We have developed a metalens based telescopic system that can correct monochromatic aberrations in FOV of 20°, whose entrance pupil diameter is 18.2 mm and metalens diameter is 46.8 mm. To the best of our knowledge, this is the largest metalens diameter (nearly 2 inches) for full-field monochromatic aberration correction meta-camera up to now. In the development process of the telescope system, the basic dimensional parameters and the optimal phases of the metalens were first solved within the framework of Chevalier Landscape lens. Then, the 2D building unit library was constructed and the overall cross-sectional structure of the concentric-ring metalens was assembled according to the optimized phase profile. After finishing the processing of metalens sample and its morphology characterization, the weak polarization dependence of the concentric-ring metalens was verified meticulously through monochromatic and orthogonal-polarized PSF test. Ulteriorly, the change in imaging contrast is quantitatively analyzed by narrowband and non-polarized PSF test. Combined with the great resolution demonstrated by the final image results, the narrowband imaging capability of the monochromatic designed system can be proved. This work comprehensively validates the feasibility of using large-diameter metalens for scene imaging and space target observation, which enables metalens telescope system to further highlight its lightweight advantage while approaching the narrowband optical performance of traditional optical lenses, thereby significantly enhancing its research value and application potential.

## Materials and methods

According to the diameter (46.8 mm), thickness (960 nm), and feature size (120 nm) of the designed concentric-ring metalens, a highly flexible and high-precision electron beam lithography (EBL) process was selected for the lithography part. The exposure equipment used is the Raith EBPG5200 electron beam lithography machine with a writing field of 1 mm, a minimum exposure feature size of 8 nm, and support for samples ranging from 1 inch to 8 inches. The large writing field with a size of 1 mm and a splicing accuracy better than 10 nm significantly enhances the processing efficiency while ensuring the processing accuracy of the metalens.

The selected photoresist is Zep520A positive resist, with a thickness of approximately 300 nm under conditions of 4000 r/min, which is adequate for transferring the 50 nm thick Chromium hard mask. Before the exposure and spin-coating of the resist, silicon nitride with a thickness of 960 nm was deposited on quartz substrate using Oxford SYSTEM 100 chemical vapor deposition (PECVD) equipment, and then chromium with a thickness of 50 nm was deposited by FHR Boxx magnetron sputtering equipment. The Deep Reactive Ion Etching (DRIE) process was chosen for the etching process, and the etching uniformity of the STS-HRM (B102) equipment used was <5%, with a side wall verticality of ≥87°.

## Supplementary information

Supplementary Information: Portable Astronomical Observation System Based on Large-Aperture Concentric-Ring Metalens
