# Supplementary material for: Portable astronomical observation system based on large-aperture concentric-ring metalens
Source: Light Sci Appl. 2025 Jan 1;14:2. doi: 10.1038/s41377-024-01656-2 (PMC11688503; doi:10.1038/s41377-024-01656-2)
Supplement: Supplementary file 1 — Supplementary Information: Portable Astronomical Observation System Based on Large-Aperture Concentric-Ring Metalens [file 41377_2024_1656_MOESM1_ESM.pdf]

Supplementary Information: Portable Astronomical Observation System Based on Large-Aperture Concentric-Ring Metalens

Jianli Wang[1,*], Yongting Deng[1,*] Chengmiao Wang[1], Yu Lin[1], Yeming Han[1], Junchi Liu[1], Xiufeng Liu[1], Hongwen Li[1], Jan G. Korvink[2,*], Yongbo Deng[2,*]

[1]Changchun Institute of Optics, Fine Mechanics and Physics, Chinese Academy of Sciences, Changchun, 130033, China;

[2]Institute of Microstructure Technology (IMT), Karlsruhe Institute of Technology (KIT), Hermann-von-Helmholtzplatz 1, Eggenstein-Leopoldshafen 76344, Germany.

*wangjianli@ciomp.ac.cn (Jianli Wang)
*dengyongting@ciomp.ac.cn (Yongting Deng)
wangchengmiao@ciomp.ac.cn (Chengmiao Wang)
linyu@ciomp.ac.cn (Yu Lin)
hanyeming@ciomp.ac.cn (Yeming Han)
liujunchi@ciomp.ac.cn (Junchi Liu)
liuxiufeng20@mails.ucas.ac.cn (Xiufeng Liu)
lihongwen@ciomp.ac.cn (Hongwen Li)
*jan.korvink@kit.edu (Jan G. Korvink)
*yongbo.deng@kit.edu (Yongbo Deng)

S1. The main performance comparison between our work and representative relevant literatures.
We have compared the main performance parameters of the metalens camera described in this paper with the relevant literature published in recent years, which mainly includes the development of single large-aperture metalens and the stop-metalens combination system. As is evident from Table. S1, our work has the advantages of both large aperture and high-resolution within wide FOV, which is of great significance to promote the wide application of the metalens telescopic system.

Reference number	Basic form	Diameter (mm)	Wavelength (μm)	Diameter-wavelength ratio	Focal length (mm)	Resolution (lp/mrad)	FOV
1	Single metalens	100	0.633	1.6e5	150	20	10″
2	Single metalens	80	1.45	5.5e4	260	15	On axis
3	Single metalens	80	10	8e3	80	6.4	On axis
4	Single metalens	50	10.6	4.7e3	34	1.8	On axis
5	Single metalens	20	1.55	1.3e4	50	2.4	On axis
6	Single metalens	20	0.66/0.53/ 0.47	3e4/3.8e4/ 4.2e4	12.9/16/18	16.5/30/35.5	On axis
7	Stop + metalens	2	0.85	2.4e3	3.36	1.3	16°
8	Stop + metalens	5.2/6.4	5.2/0.94	1e3/6.8e3	2/2.5	0.14/0.57	＞170°
This work	Stop + metalens	46.8	0.633	7.4e4	91.5	7.3	20°

Table. S1. Main parameter list of representative relevant literatures.

Reference in Table S1:

1. Park, J.S. et al. All-glass 100 mm Diameter Visible Metalens for Imaging the Cosmos. *ACS Nano* **18**, 3187-3918 (2024).
2. Zhang, L. D. et al. High-Efficiency, 80 mm Aperture Metalens Telescope. *Nano Lett.* **23**, 51-57 (2023).
3. Li, J. W. et al. Largest aperture metalens of high numerical aperture and polarization independence for long-wavelength infrared imaging. *Opt. Express* **30**, 28882-28891 (2022).
4. Hou, M. M., Chen, Y., Li, J. Y. & Yi, F. Single 5-centimeter-aperture metalens enabled intelligent lightweight mid-infrared thermographic camera. *Sci. Adv.* **10**, eado4847 (2024).
5. She, A., Zhang, S. Y., Shian, S., Clarke, D. R., & Capasso, F. Large area metalenses: design, characterization, and mass manufacturing. *Opt. Express* **26**, 1573-1585 (2018).
6. Lee, G. Y. et al. Metasurface eyepiece for augmented reality. *Nat. Commun.* **9**, 4562 (2018).
7. Engelberg, J. et al. Near-IR wide-field-of-view Huygens metalens for outdoor imaging applications. *Nanophotonics* **9**, 361-370 (2020).
8. Shalaginov, M. Y. et al. Single-Element Diffraction-Limited Fisheye Metalens. *Nano Lett.* **20**, 7429-7437 (2020).

S2. The specific design process and results of the combined telescopic system with aperture stop and metalens.

In this study, Zemax OpticStudio software was employed to optimize the basic dimensional parameters of the meta-camera and the modulated phase of the metalens. The target FOV for the meta-camera is 20°, and the diameter of the metalens is slightly less than 2 inches. The detector used is OR-400BSI-PRO high-frame-rate sCMOS camera (Pixel resolution of 2048×2048, pixel pitch D_{pixel} =11μm) from Changchun Changguang Aorun photoelectric Technology Co., LTD. Under consideration of distortion, the image field corresponding to a 10° incident light is set to match the diagonal size of the detector, thus the focal length of the system is 91.5mm. An initial structure of the Chevalier Landscape lens that can achieve near-diffraction limit focusing is obtained, as shown in Fig. S1(a). Within range where the spatial frequency is less than the cut-off frequency ($1/2D_{pixel}$ = 46lp/mm) of the detector, the MTF curve of the initial structure is shown in Fig. S1 (b).

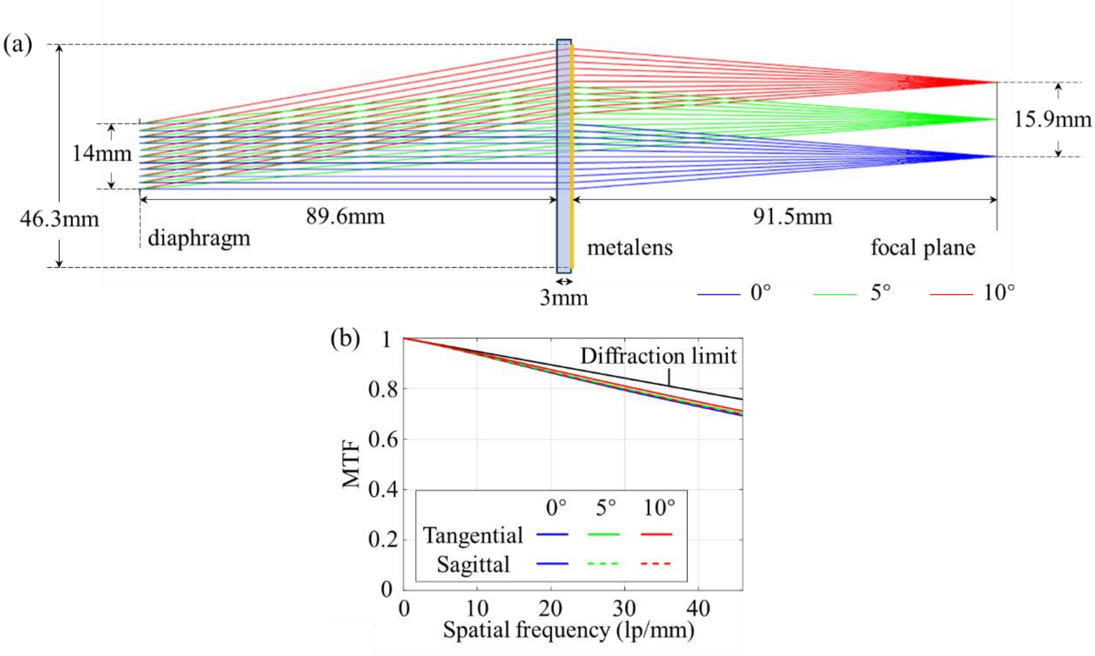

Fig. S1. (a) The initial 2D structure of the optical system; (b) MTF curve of the initial optical system.

The initial structure exhibits excellent resolution (MTF ≈ 0.7@46lp/mm), but such high resolution may be redundant for the detector. This study assumes that the optimal resolution match between the focusing system and the detector occurs when MTF of the focusing system is 0.4 at the cut-off frequency of the detector. Therefore, the resolution requirements for the focusing system can be appropriately lowered. On the other hand, the entrance pupil diameter of the optical system plays a pivotal role in determining the maximum observable stellar magnitude facing the observation requirements for faint celestial objects in space. Therefore, building upon the initial optical system, the in-depth optimization was conducted with the even-order phase coefficients of the metalens and the distance between the stop and the metalens as variables in the additional variable space released by relaxing the MTF demands. The primary optimization goal is to maximize the entrance pupil diameter. The final optical system is depicted in Fig. S2.

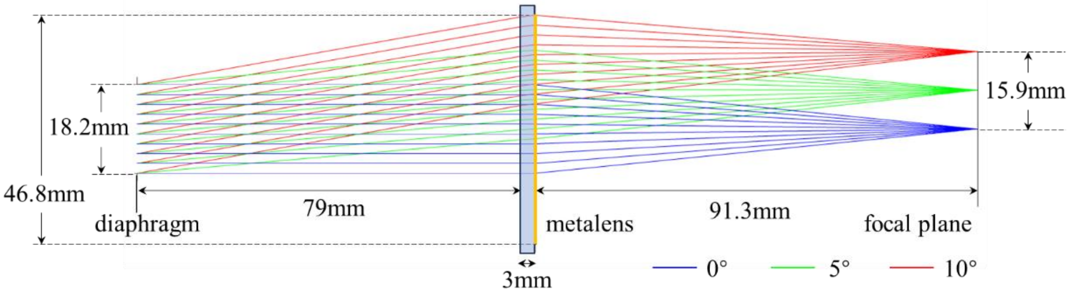

Fig. S2. The final 2D structure of the optical system.

The final optical system features a luminous flux 1.7 times that of the initial structure, significantly enhancing the detection capability for faint celestial objects in space. Correspondingly, the optical path slightly deviates from the image-side telecentricity, but the PSF and the MTF still exhibit a high degree of similarity across different fields of view. The mean MTF for different FOVs tends to be 0.4@46lp/mm, ensuring the resolution match between the focusing system and the detector. The optimized phase coefficients of the metalens are shown in Table. S2.

s	1	2	3	4	5	6
a_s	-2.968e4	-154.850	626.015	-1072.277	826.903	-236.108

Table. S2. The final optimization results of the even-order phase coefficient of the metalens.

S3. Optimization of underlying parameters based on maximization of focusing efficiency.

To determine the optimal unit width (Λ) and structural height (H), the light intensity distributions in focal plane and the corresponding focusing efficiencies are calculated for the metalens constructed with different underlying parameters Λ and H. Taking into account the existing processing technology for Si_3N_4 material, the minimum width of nano-ring and its gap, that is, the feature size (w_{min}), is initially constrained to be no less than 120nm. Under this constraint, the sampling range for Λ is from the minimum width allowing the existence of double-ring structure ($4w_{min}$ = 480nm) to a design wavelength λ (approximately 630nm). The sampling range for H is from $\lambda/(n\text{-}1) \approx$ 600nm to the maximum height ($12w_{min}$ = 1440nm) that can be supported by limiting the aspect ratio ≤12, where n is the refractive index of Si_3N_4. The sampling interval for both are set at 30nm.

After arranging the nano-structures and simulating the light intensity in focal plane, the focusing efficiency for specific underlying parameters can be calculated. The diffractive focusing efficiency is defined as the average ratio of the focal spot energy to the near-field emitted energy of the metalens at different fields of view, as shown in Fig. S3(a). The global focusing efficiency is defined as the average ratio of the focal spot energy to the incident energy on the metalens at different fields of view, as shown in Fig. S3(b). The focal spot energy is defined as the energy density integral within five times the half-width of the diffraction-limited focal spot. To suppress the decrease in imaging contrast caused by undesired diffractive orders, we take the maximization of diffraction focusing efficiency as the primary goal and select Λ = 600nm and H = 960nm as the final underlying parameters of actual processed metalens. Throughout the main text and other sections of the Supplementary Information, the focusing efficiency mentioned, unless otherwise specified, refers to the diffractive focusing efficiency.

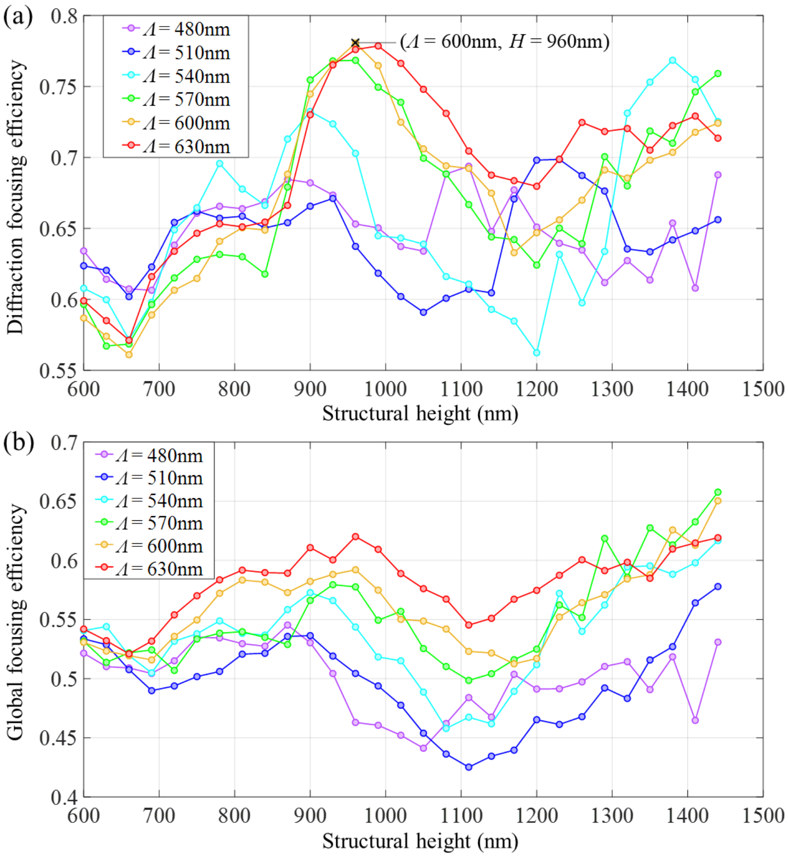

Fig. S3. Focusing efficiency of the metalens with different underlying parameters;
(a) Diffraction focusing efficiency; (b) Global focusing efficiency.

Additionally, it should be noted that each data point in Fig. S3 represents a single simulated metalens.

To compress the computational burden generated by traversing a large number of underlying parameter pairs, the focusing efficiency presented here is based on the 1D arrangement of nanostructures (1D focusing lens). Consequently, the focusing efficiency is relatively higher compared to an actual concentric-ring metalens. The purpose of this simulation is to retrieve the optimal set of underlying parameters to obtain a unit library with modulation phase covering 2π as much as possible and high transmittance. These optical properties of the unit library have little substantial relation with the dimension of the structure arrangement, so this approximation of dimensionality reduction is reasonable.

S4: Simulation details of the light intensity distribution in focal plane

Due to the polarization-dependent light modulation of the strip-shaped nanostructures in a local perspective, the simulation calculation of the light firld distribution in focal plane under non-polarized light incidence requires separate consideration of incoherent x- and y-polarized light. Fig. S4 illustrates the schematic diagram of preprocessing the complex amplitudes for different polarizations before calculating the light field distribution in focal plane, taking the example of y-polarized light at a 10° oblique incidence along the y-axis. Firstly, the projection area of the incident light on the metalens is determined according to the incident angle, and the discrete sampling is carried out in which the interval is equal to the unit width Λ. For any sampling point, the polar coordinates (r, α) relative to the center of the metalens is used to determine which ring-zone it is located in, and the specific position of the sampled point within the ring-zone is ignored. The nano-ring modulates the incident x- or y-polarized light along the radial and tangential directions, and the complex transmittances in both directions are given by the obtained optical field simulation results of the corresponding 2D unit. The modulated light is decomposed and superimposed again in Cartesian coordinates to obtain the near-field output complex amplitude for x- and y-polarizations, as expressed in Eq. (S1), where $\tilde{T}_r$ and $\tilde{T}_t$ represent the complex transmittance of the nano-ring for incident light polarized along the radial and tangential directions, corresponding to $\tilde{T}_{\mathrm{TM}}$ and $\tilde{T}_{\mathrm{TE}}$ in the 2D unit simulation results, respectively. Similarly, the near-field output complex amplitude of x-polarized incident light is shown in Eq. (S2).

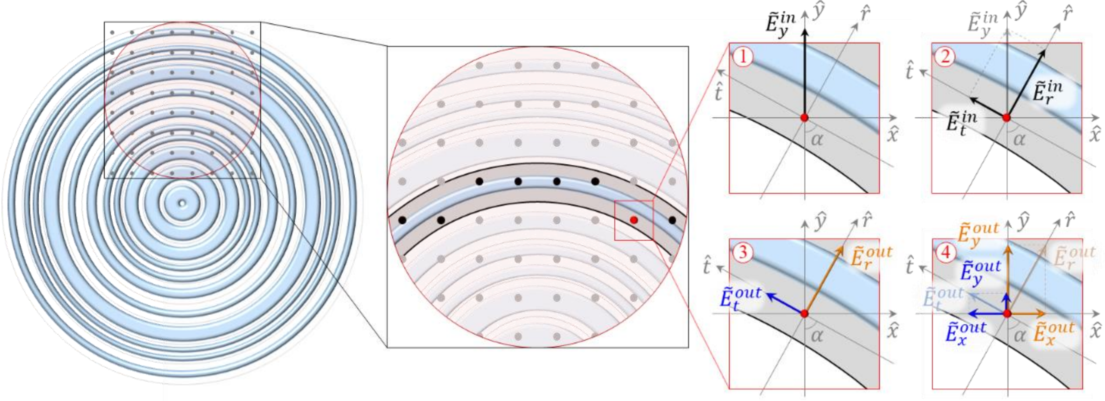

Fig. S4. Schematic diagram of discrete sampling on the metalens surface and the calculation of the output complex amplitude for the oblique incident light with y-polarization.

$$\tilde{E}_x^{\text{out}} = (\tilde{T}_r - \tilde{T}_t)\cdot\sin\alpha\cos\alpha\cdot\tilde{E}_y^{\text{in}}$$
$$\tilde{E}_y^{\text{out}} = (\tilde{T}_r\sin^2\alpha + \tilde{T}_t\cos^2\alpha)\cdot\tilde{E}_y^{\text{in}} \tag{S1}$$

$$\tilde{E}_x^{\text{out}} = (\tilde{T}_r\cos^2\alpha + \tilde{T}_t\sin^2\alpha)\cdot\tilde{E}_x^{\text{in}}$$
$$\tilde{E}_y^{\text{out}} = (\tilde{T}_r - \tilde{T}_t)\cdot\sin\alpha\cos\alpha\cdot\tilde{E}_x^{\text{in}} \tag{S2}$$

According to the theory of angular spectrum diffraction, the light field distributions of x-polarized and y-polarized incident light diffracted from the near-field exit plane to the focal plane are calculated separately, as shown in Eq. (S3), where $\tilde{E}_{x,y}^{out}(x, y)$ and $\tilde{E}_{x,y}^{f}(x, y)$ represent x-,y-polarized light field distribution in the near-field exit plane and the focal plane, respectively; f_x, f_y are the spatial frequencies corresponding to x, y spatial coordinates; $\mathscr{F}$ and $\mathscr{F}^{-1}$ represent the Fourier transform and its inverse transformation, respectively. The focusing efficiency corresponding to the obtained light intensity distribution is used to guide the selection of the underlying parameters Λ and H, and the phase difference constant $\Delta\varphi$. It should be noted that the above calculation of the light field distribution in focal plane is based on scalar diffraction theory, rather than Maxwell full-wave simulation, which is extremely difficult to achieve for such a large aperture. Therefore, this method neglects the coupling between the nano-rings,

but it can be verified that this reasonable approximation has almost no effect on the relative intensity distribution of the focal spot.

$$\tilde{E}^{f}_{x,y}(x,y)=\mathscr{F}^{-1}\left\{\mathscr{F}\left[\tilde{E}^{out}_{x,y}(x,y)\right]\cdot\exp\left(\frac{2\pi i}{\lambda}f\cdot\sqrt{1-(\lambda f_x)^2-(\lambda f_y)^2}\right)\right\} \tag{S3}$$

For the established unit settings with Λ = 600nm and H = 960nm, the focusing efficiency curves for different values of $\Delta\varphi$ are shown in Fig. S5. The optimal value for $\Delta\varphi$ is determined to be -0.09π, corresponding to a focusing efficiency of 65%. The focusing efficiency level maybe slightly lower than that of monochromatic building-block-array metalens, which is essentially caused by the unequal complex transmissions of radial (TM) and tangential (TE) polarization in the concentric ring structure. The concentric ring structure slightly sacrifices focusing efficiency, which has almost no impact on imaging clarity, but effectively improves the processability and mechanical stability of the structure, making it more practical for the application of large-aperture metalenses. The light intensity distributions in focal plane for different incident angles are shown in Fig. S6. All subplots are normalized based on the results of non-polarized light incidence at the corresponding incident angles. The simulation ranges are all 40μm×40μm.

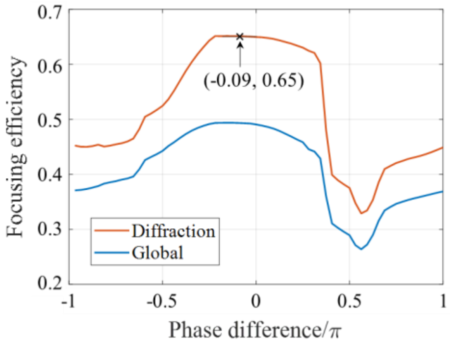

Fig. S5. Diffraction focusing efficiency and global focusing efficiency curves for different $\Delta\varphi$ values.

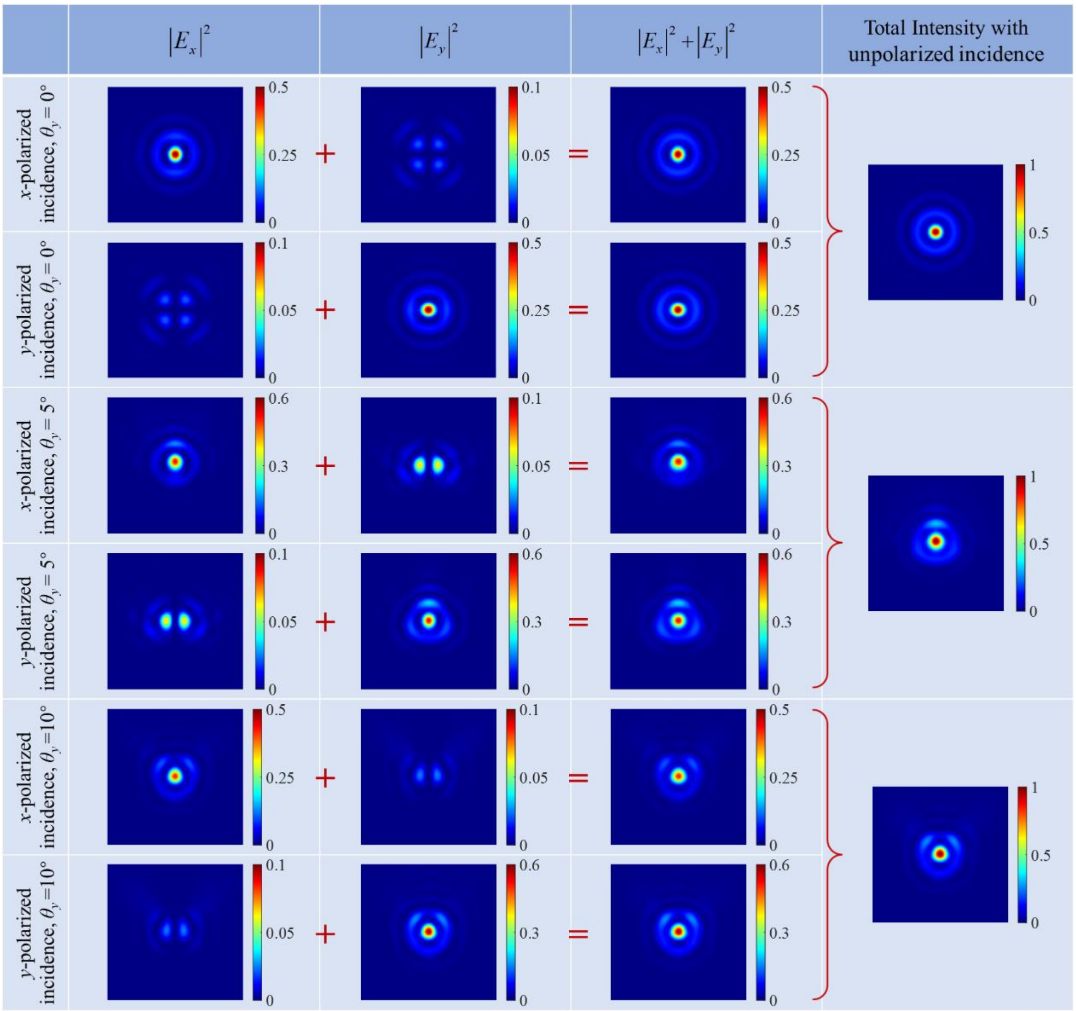

Fig. S6. The distributions of light intensity in focal plane for different incident angles.

It can be observed that there is orthogonal polarization conversion in the outgoing light, but the intensity component of the polarization conversion is nearly an order of magnitude smaller than that without polarization conversion, almost having no impact on the imaging quality. When the light is incident with different polarizations, there will be slight differences in the intensity distribution within the focal plane, which is because the nano-ring has a higher focusing efficiency for radially polarized light compared to tangentially polarized light, that is, the phase modulation range of the nano-ring for the TM wave in its 2D cross-section is larger than that of the TE wave. This characteristic gives rise to a series of detailed features that are not present in strictly polarization-independent lens focusing. For the normal incident light with x-polarization, the side lobes along the x-axis in the focal spot are slightly weaker than those along the y-axis. This is because the x-polarized incident light is radially polarized along the x-axis and tangentially polarized along the y-axis. Thus the concentric-ring structure results in higher focusing efficiency along the x-axis compared to the y-axis. Meanwhile, at an incidence angle of $\theta_y = 10°$, the intensity of the focal spot for x-polarized light is slightly smaller than that for y-polarized light because the tangentially polarized component dominates for x-polarized light, while the radially polarized component dominates for y-polarized light, which also confirms the qualitative conclusions mentioned above. However, the deviation in intensity distribution of the focal spots for the different polarization states is much smaller than the intensity of the focal spot itself. Therefore, the concentric-ring metalens in this study can still be considered as polarization-insensitive devices from a global perspective.

S5: Measuring details for the relative intensity of the first-order side lobes of the PSFs.

In the testing process of the relative intensity of the first-order side lobes, the direct measurement results of the side lobe intensity distributions did not align well with simulations. Upon further analysis, we identified an unobservable factor affecting the side lobe morphology: a lateral alignment error of approximately 0.5mm between the aperture stop and the metalens, which is a residual issue encountered during the integration of the two into a meta-tube. The magnitude and orientation of this lateral alignment error were estimated and the corresponding simulation was conducted. The results showed that since the alignment error is taken into account, the simulated PSF was more similar to the measured results compared to those in Fig. S6, as shown in Fig. S7, which largely corroborates our hypothesis.

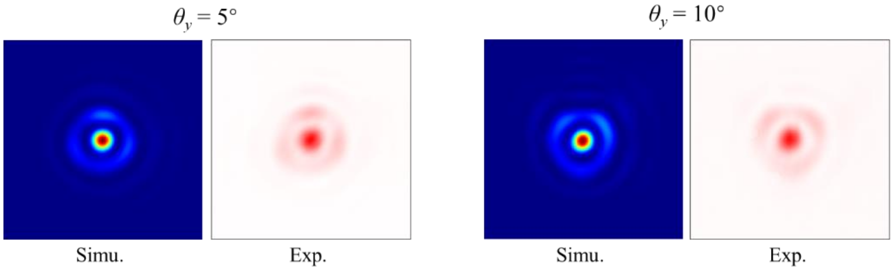

Fig. S7. The comparison between the simulated and measured PSF considering the alignment error under the condition of non-polarized oblique incidence.

To eliminate this effect, the side lobe intensity measurements of non-polarized incidence was used to correct those of the x- and y-polarization incidence. More specifically, for the three cases of x-, y- and non-polarized light, the maximum side lobe intensities were found at each azimuth β, denoted as $I_x^{e0}(\beta)$, $I_y^{e0}(\beta)$, $I_u^{e0}(\beta)$, and the corresponding simulation intensity is $I_x^s(\beta)$, $I_y^s(\beta)$, $I_u^s(\beta)$, respectively. In the actual data processing, the side lobe intensity deviation of the non-polarized PSF caused by alignment error is regarded as the standard value of error correction, and the side lobe intensity of x- and y-polarization is corrected to $I_x^{e1}(\beta)$ and $I_y^{e1}(\beta)$ accordingly, as shown in Eq. (S4). The intensity correction result of the side lobes is shown in Fig. S8. All side lobe intensities mentioned here have been linearly scaled, that is, the mean square values of the intensities are always 1 and the area enclosed by the closed intensity curves in the polar diagrams are always π.

$$\begin{aligned} I_x^{e1}(\beta) &= I_x^{e0}(\beta)/I_u^{e0}(\beta)\times I_u^{s}(\beta) \\ I_y^{e1}(\beta) &= I_y^{e0}(\beta)/I_u^{e0}(\beta)\times I_u^{s}(\beta) \end{aligned} \tag{S4}$$

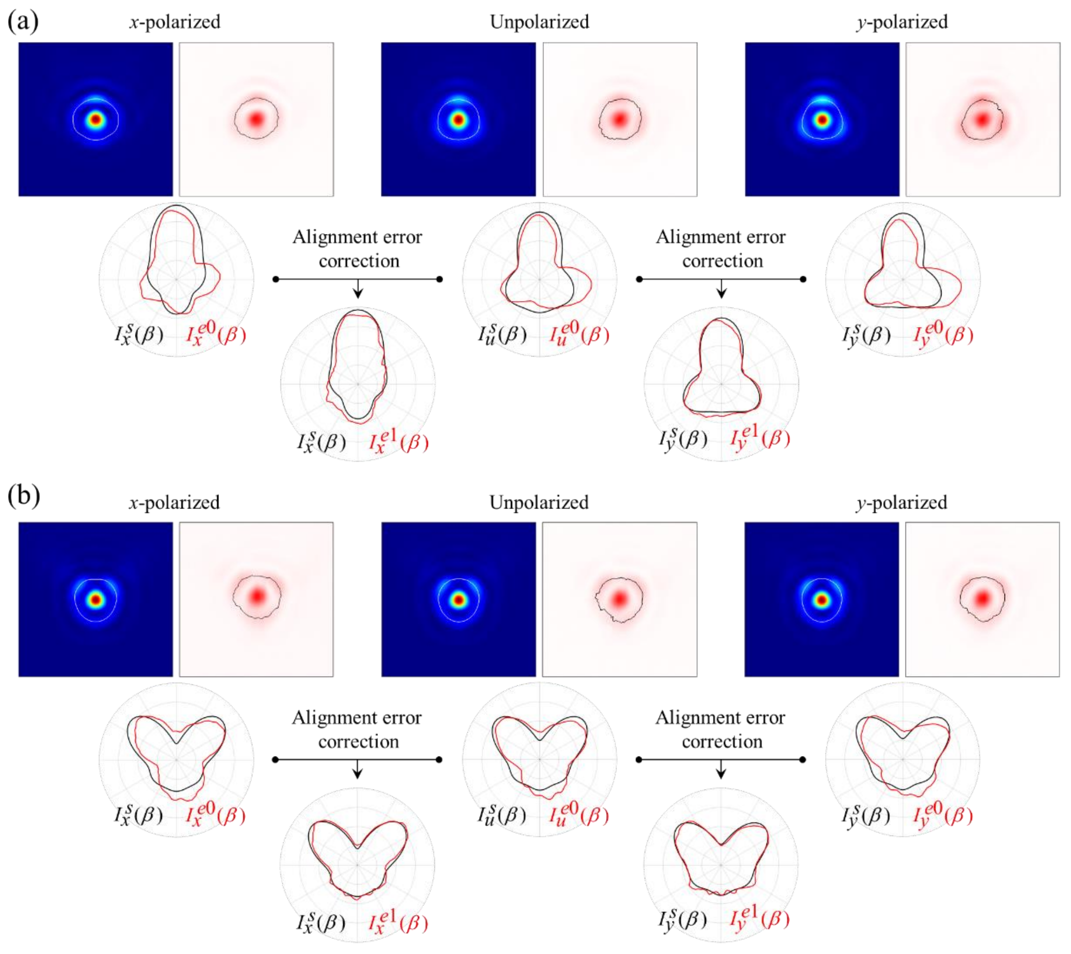

Fig. S8. Comparison diagram of experimental and simulated results of PSF and their side lobe intensities, where the solid line in PSF diagram represent the position of the maximum intensity point under each azimuth angle. In polar diagrams of relative side lobe intensities, black lines represent simulation results, and red lines represent testing results before and after alignment error correction. The radial coordinate ranges are all $r\in[0,2]$. (a) θ_y=5°; (b) θ_y=10°.

It can be seen from Fig. S8 that the corrected side lobe intensity distribution of x- and y-polarization closely match the simulated intensity distribution. Even under the condition that the side lobe intensity is insensitive to the incident polarization state in a 10 ° oblique incidence, the polarization state of the measured results can still be distinguished based on the subtle differences between the simulated PSFs. The above analysis thoroughly validates the inherent connection between the side lobe intensity and incident polarization states, thereby demonstrating the rationality of the optical field calculation method based on polarization decomposition and conversion.
